# Prognostic Value of the Preoperative ALBI Score for Postoperative Bleeding After Isolated CABG

**DOI:** 10.3390/jcm15218370

**Published:** 2026-10-27

**Authors:** Alper Özbakkaloğlu, Önder Turgut Bozkurt, Dağlar Cansu, Enver Oğuzhan Metehan, Tufan Erdem Emeklikol, Safa Göde, Şahin Bozok

**Affiliations:** 1 Department of Cardiovascular Surgery, İzmir Bakırçay University, Menemen 35665, Turkey; 2 Department of Cardiovascular Surgery, İstanbul Mehmet Akif Ersoy Thoracic and Cardiovascular Surgery Hospital, Istanbul 34303, Turkey

**Keywords:** coronary artery bypass grafting, Albumin–Bilirubin score, postoperative bleeding

## Abstract

**Background**: The Albumin–Bilirubin (ALBI) score is an easy-to-use, objective marker of liver synthetic function that has demonstrated prognostic value in various cardiovascular conditions. However, its role in predicting postoperative bleeding after CABG has not been previously investigated. **Methods**: Patients were divided into two groups according to their median preoperative ALBI score (−2.97): Low ALBI (n = 78) and High ALBI (n = 79). The primary outcome was excessive postoperative bleeding, defined as cumulative drainage exceeding 1000 mL within the first 24 postoperative hours. Secondary outcomes included transfusion requirements, mechanical ventilation duration, and length of hospital stay. Correlation and regression analyses were performed to assess the relationship between the ALBI score and postoperative bleeding. **Results**: The High ALBI group demonstrated a significantly greater postoperative drainage volume (450 [350–700] mL vs. 350 [300–487.5] mL, *p* = 0.007), higher erythrocyte transfusion (*p* = 0.001) and fresh frozen plasma transfusion requirements (*p* = 0.034), and prolonged mechanical ventilation duration (*p* < 0.001) and hospital stay (*p* = 0.003) compared to the Low ALBI group. Spearman correlation analysis revealed a significant positive correlation between the ALBI score and drainage volume (rho = 0.272, *p* < 0.001), and thus the ALBI score was identified as a significant predictor of excessive postoperative bleeding exceeding 1000 mL (OR: 5.93, 95% CI: 1.63–21.60, *p* = 0.007). **Conclusions**: The preoperative ALBI score is an easy-to-use and readily available tool that may serve as an effective adjunct for identifying patients at an elevated risk of postoperative bleeding prior to isolated CABG.

## 1. Introduction

Coronary artery bypass grafting (CABG) is one of the most frequently performed cardiac surgical procedures and is generally associated with low mortality and morbidity, particularly in low-risk patients [1]. However, postoperative bleeding remains a relevant clinical concern [2].

The effectiveness of patient blood management depends not only on the correction of preoperative anemia and the appropriate management of antithrombotic therapy but also on accurate preoperative risk stratification to detect patients at high risk of significant blood loss [3].

The coagulation process is closely linked to liver function, which is responsible for the synthesis of most coagulation factors except calcium [4]. Even slight impairment of the hepatic synthetic function may lead to significant hemostatic imbalance, particularly in settings such as cardiopulmonary bypass (CPB), where coagulation factors are consumed [5,6].

The Albumin–Bilirubin (ALBI) score was originally described by Johnson et al. in 2015 as an objective, simple marker of hepatic synthetic function and was initially introduced into clinical practice to evaluate liver function in patients with hepatocellular carcinoma [7]. A high ALBI score is a strong independent predictor of adverse outcomes across various cardiovascular conditions, including heart failure, heart valve surgery, and transcatheter aortic valve implantation [8,9,10]. However, the ALBI score’s role in predicting postoperative bleeding and drainage volume after CABG remains insufficiently investigated. Various scoring systems for bleeding risk have been developed for cardiovascular surgery, such as the Papworth and Will-Bleed scores, but most of these models are based on complex scoring systems that do not directly assess mild synthetic dysfunction in the liver [11,12].

The aim of this study is to investigate the prognostic value of the preoperative ALBI score in predicting postoperative drainage amounts in patients undergoing isolated CABG, which would provide a simple and cost-effective adjunct for preoperative risk assessment.

## 2. Materials and Methods

### 2.1. Study Population

**Figure 1:**
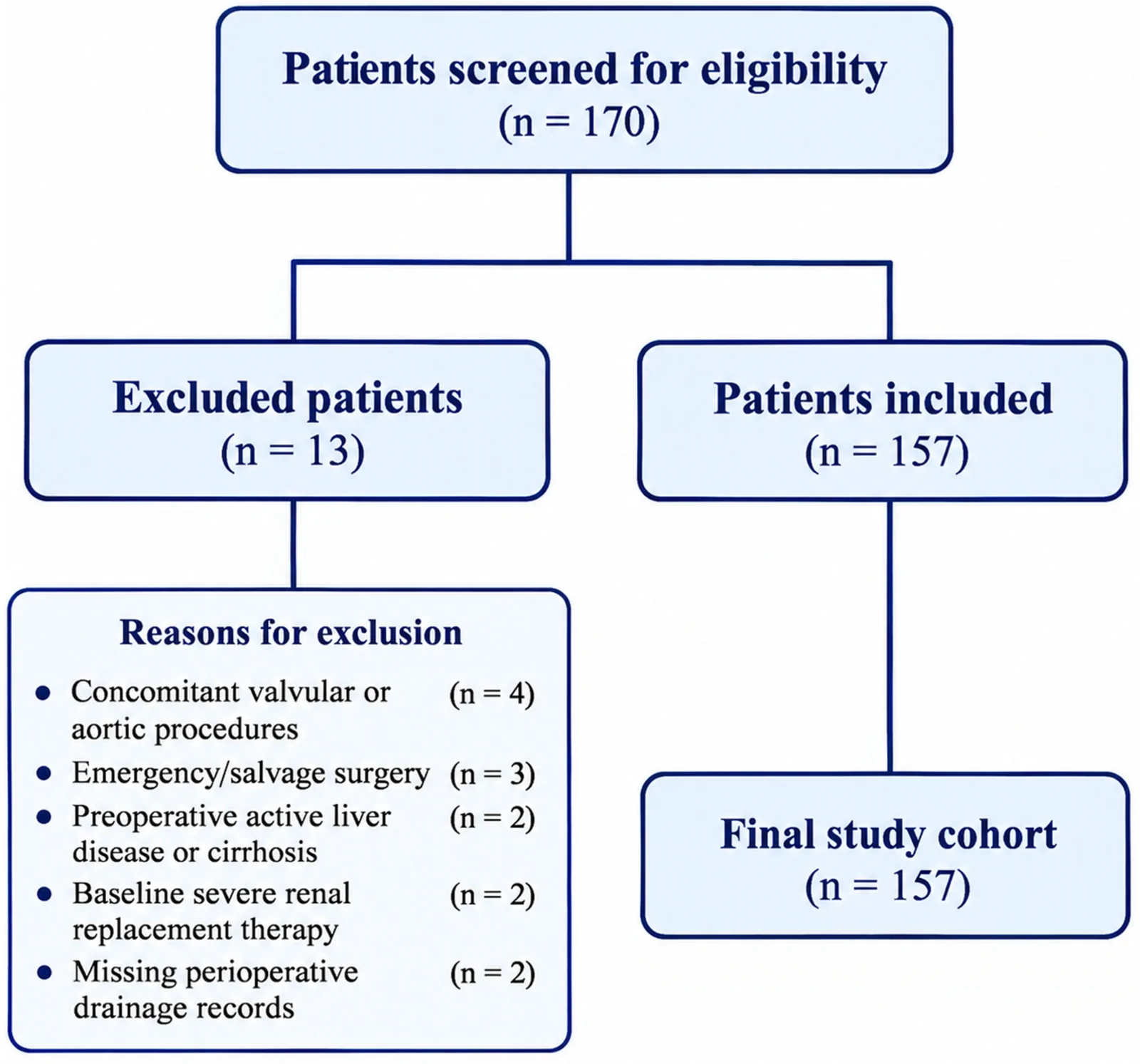
Flow diagram of patient selection.

This retrospective observational cohort study included consecutive patients who underwent isolated CABG between June 2021 and January 2024 at our tertiary center. The inclusion criteria were as follows: having undergone isolated CABG surgery and complete preoperative laboratory results, including serum albumin and total bilirubin levels, within 24 h prior to surgery. The exclusion criteria comprised having undergone concomitant valvular or aortic procedures and/or emergency/salvage operations, having preoperative active liver disease or cirrhosis, having undergone baseline severe renal replacement therapy, and missing perioperative drainage records. Thirteen patients were excluded because they met at least one of these criteria. Consequently, a total of 157 patients who underwent surgery were included in this study (Figure 1). Of these 157 patients, one patient had previously undergone surgical repair of an atrial septal defect (ASD). Isolated CABG was performed on this patient during the study period without any concomitant cardiac procedure.

### 2.2. Data Collection

Pre- and postoperative data were obtained from the institution’s electronic database. Preoperative data included the EuroSCORE II, left ventricular ejection fraction, and currently used anticoagulant or antiplatelet medications. Preoperative laboratory values were obtained from blood samples collected within 24 h prior to surgery. Serum albumin and total bilirubin levels were obtained from routine venous blood samples drawn at a standardized time point within 24 h prior to surgery and were used to calculate the preoperative ALBI score. Crucially, all blood samples were collected prior to anesthesia induction, systemic heparinization, cardiopulmonary bypass initiation, and the administration of any preoperative intravenous albumin infusion or blood product transfusions. Recorded intraoperative variables included surgical techniques (with and without CPB), CPB duration, and aortic clamping durations. Acute renal failure not requiring dialysis was defined according to the following basic criteria: (i) serum creatinine level ≥ 1.5 times higher than baseline, (ii) serum creatinine level ≥ 0.3 mg/dL (≥26.4 mmol/L), or (iii) urine output < 0.5 mL/kg/hour for more than 6 h. Early postoperative outcomes, including data such as length of stay in the intensive care unit and hospital, total postoperative drainage volume, and need for blood product transfusions, were collected for analysis. The formula used to calculate the ALBI score is as follows: (albumin × −0.085) + (log10 bilirubin × 0.66), where albumin is measured in g/L and bilirubin in μmol/L, as previously described in the literature.

### 2.3. Study Design

The study population was divided into two cohorts based on the preoperative median ALBI score (−2.97). Patients scoring greater than this threshold were assigned to the High ALBI group (n = 79), while those scoring less than or equal to −2.97 were assigned to the Low ALBI group (n = 78). The primary outcome of this study was excessive postoperative bleeding, defined as cumulative mediastinal and chest tube drainage exceeding 1000 mL within the first 24 postoperative hours. Secondary outcomes included continuous total postoperative drainage volume (mL), requirement for and quantity (units) of postoperative erythrocyte transfusion (ERT) and fresh frozen plasma (FFP) transfusion, intensive care unit (ICU) length of stay (hours), and total postoperative hospital stay (days). In accordance with institutional protocol, dual antiplatelet therapy or P2Y12 inhibitors were routinely discontinued at least 5 days prior to elective CABG surgery, while aspirin was maintained until the day of operation. In the operating room, heparin was administered at a dose of 400 IU/kg to ensure optimal intraoperative management and hemostatic control, with a target activated clotting time (ACT) of >480 s. Following the completion of anastomoses, systemic heparin was neutralized with protamine at a 1:1 ratio. If the post-protamine ACT remained above 150 s, an additional 5000 IU of protamine was administered, aiming to achieve a post-reversal ACT of <150 s. Furthermore, regarding antifibrinolytic management, our center routinely implements a standardized intraoperative protocol consisting of an initial 1 g tranexamic acid bolus followed by a continuous intravenous infusion of 7.5 mg/kg/hour prior to chest closure and drain placement. In our clinic, standardized antifibrinolytic protocol is utilized postoperatively in the intensive care unit.

### 2.4. Statistical Analysis

Statistical analyses were performed using R version 4.0.3 (R Foundation for Statistical Computing). Continuous variables were evaluated for normality utilizing the Shapiro–Wilk test, while normally distributed data were analyzed using the independent-samples Student’s *t*-test and non-normally distributed data were analyzed using the Mann–Whitney U test. Categorical data were compared between the two groups using the Chi-square test or Fisher’s exact test, where appropriate. The correlation between the ALBI score and total postoperative drainage volume was evaluated using the Spearman correlation coefficient. Univariate and multivariate logistic regression analyses were performed to identify the independent predictors of excessive postoperative bleeding (defined as bleeding exceeding 1000 mL). Odds ratios (ORs) and 95% confidence intervals (CIs) were calculated for each variable. To reduce the risk of overfitting due to the limited number of outcome events (n = 9), multivariate logistic regression was restricted to only two candidate estimators. The discriminative ability of the preoperative ALBI score for predicting excessive postoperative bleeding (>1000 mL) was evaluated using receiver operating characteristic (ROC) curve analysis and the area under the curve (AUC) with 95% confidence intervals (CIs). No statistical imputation techniques (such as mean imputation or multiple imputation) were applied, thereby preventing artificial reductions in variance or imputation-induced estimation bias. *p*-values < 0.05 were considered statistically significant.

## 3. Results

A total of 157 patients were evaluated and stratified into two groups based on their preoperative scores: the Low ALBI group (n = 78) and the High ALBI group (n = 79). There were no statistically significant differences regarding age, sex distribution, body mass index, comorbidities (including diabetes mellitus, hypertension, hyperlipidemia, COPD, and peripheral and carotid artery diseases), and surgical characteristics, such as left ventricular ejection fraction, redo cardiac surgery, urgent surgery rates, EuroSCORE II, and preoperative anticoagulant/antiplatelet medication use (*p* > 0.05). Regarding laboratory parameters, the ALBI score (−3.29 ± 0.23 vs. −2.61 ±0.35, *p* < 0.001), albumin levels (44.92 ± 3.09 g/L vs. 38.49 ± 4.02 g/L, *p* < 0.001), and total bilirubin levels (0.48 [0.31–0.58] mg/dL vs. 0.62 [0.41–0.92] mg/dL, *p* < 0.001) significantly differed between the Low and High ALBI groups. Hematocrit levels were significantly higher in the Low ALBI group compared to the High ALBI group (40.31 ± 4.88 vs. 38.22 ± 5.18, *p* = 0.014). No statistically significant differences were observed for the remaining laboratory markers, including creatinine, glomerular filtration rate, INR, ALT, AST, and C-reactive protein (*p* > 0.05). The preoperative baseline and laboratory characteristics of the study population are presented in detail in Table 1.

Regarding intraoperative characteristics, there were no statistically significant differences between the Low and High ALBI cohorts in terms of off-pump techniques (8 (10.3%) vs. 3 (3.8%), *p* = 0.130). Similarly, no significant variations were observed between the groups regarding CPB time (102 ± 37 min vs. 114 ± 50 min, *p* = 0.112) and cross-clamp time (68 ± 25 min vs. 69 ± 23 min, *p* = 0.721).

There were no statistically significant differences between the Low and High ALBI groups regarding the incidence of major postoperative complications, including acute kidney injury stratified by dialysis and non-dialysis requirements (*p* = 0.785), 30-day mortality (2.6% vs. 8.9%, *p* = 0.167), and re-exploration for bleeding (*p* = 0.999). The median total drainage volume was significantly lower in the Low ALBI cohort than in the High ALBI cohort (350 [300–487.5] mL vs. 450 [350–700] mL, *p* = 0.007). Patients in the High ALBI group required a significantly higher volume of blood product transfusion, including total erythrocyte transfusion (ERT) units (2 [1–3] vs. 1 [0–2], *p* = 0.001) and total fresh frozen plasma (FFP) units (2 [1–4] vs. 2 [1,2], *p* = 0.034). The median duration of postoperative mechanical ventilation was significantly shorter in the Low ALBI group compared to the High ALBI group (7 [5.75–10] hours vs. 10 [7–13.75] hours, *p* < 0.001). A comparison of the postoperative outcomes and clinical characteristics is summarized in detail in Table 2.

Among the 157 patients, nine (5.7%) developed postoperative excessive bleeding exceeding 1000 mL. Of these nine excessive bleeding events, six (66.7%) occurred in the High ALBI group, whereas three (33.3%) occurred in the Low ALBI group. Univariate logistic regression analysis was performed to identify the predictors of excessive bleeding. As shown in Table 3, the ALBI score was significantly associated with the outcome (OR: 5.93, 95% CI: 1.63–21.60, *p* = 0.0069) and cardiopulmonary bypass (CPB) time (OR: 1.01, 95% CI: 1.00–1.03, *p* = 0.029). Other variables, including ASA use, gender, EuroSCORE II, and hypertension, were not statistically significant. In the multivariable logistic regression model adjusted for CPB duration, the preoperative ALBI score remained an independent predictor of excessive postoperative bleeding (OR: 7.80, 95% CI: 1.87–32.50, *p* = 0.0048).

**Figure 2:**
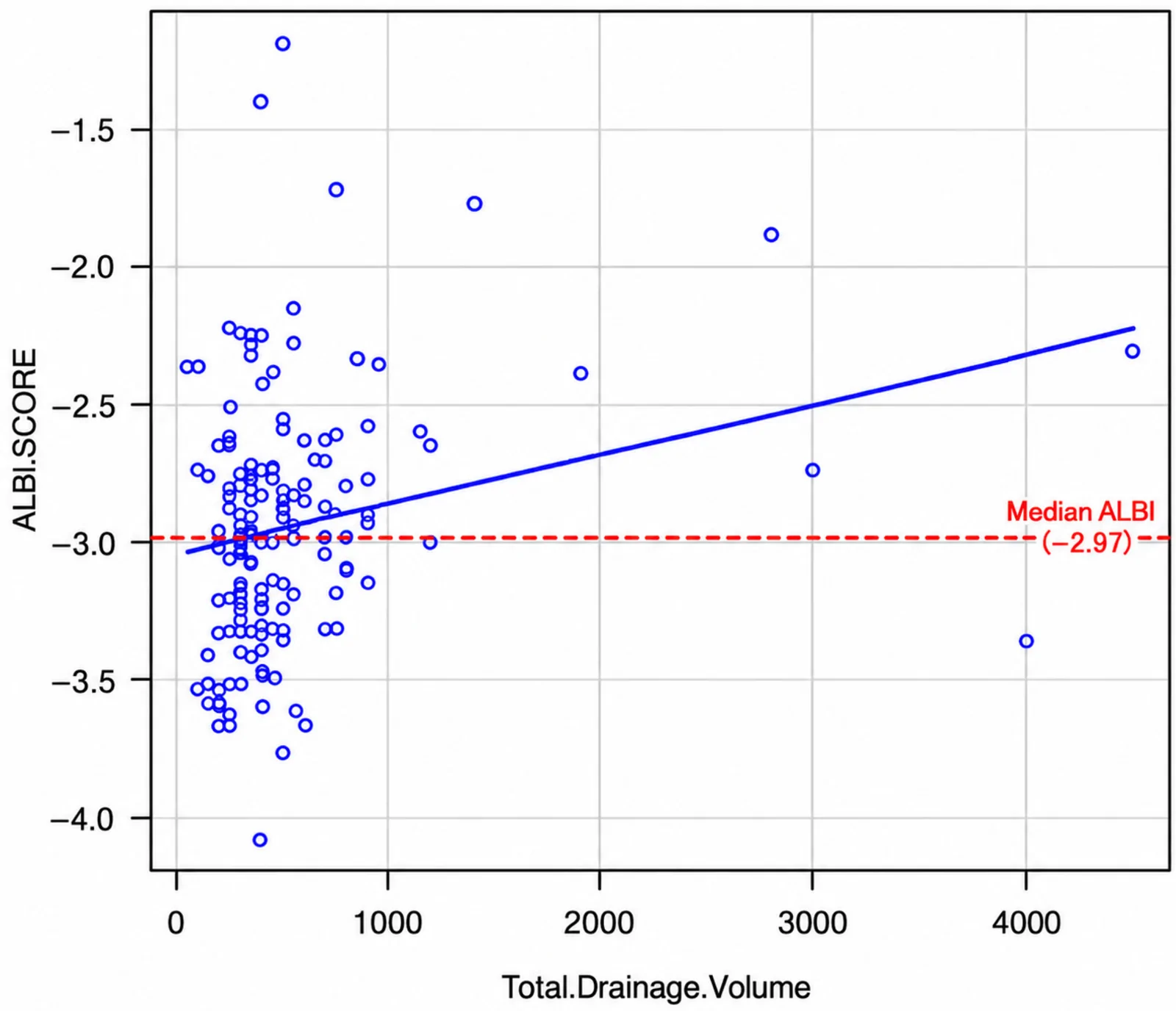
Correlation between the preoperative Albumin–Bilirubin (ALBI) score and total postoperative drainage volume. Spearman correlation analysis demonstrated a significant positive correlation (rho = 0.272, *p* < 0.001).

Spearman correlation analysis demonstrated a statistically significant positive correlation between the ALBI score and total postoperative drainage volume (rho = 0.272, *p* < 0.001); high ALBI scores were associated with increased postoperative drainage volumes (Figure 2).

**Figure 3:**
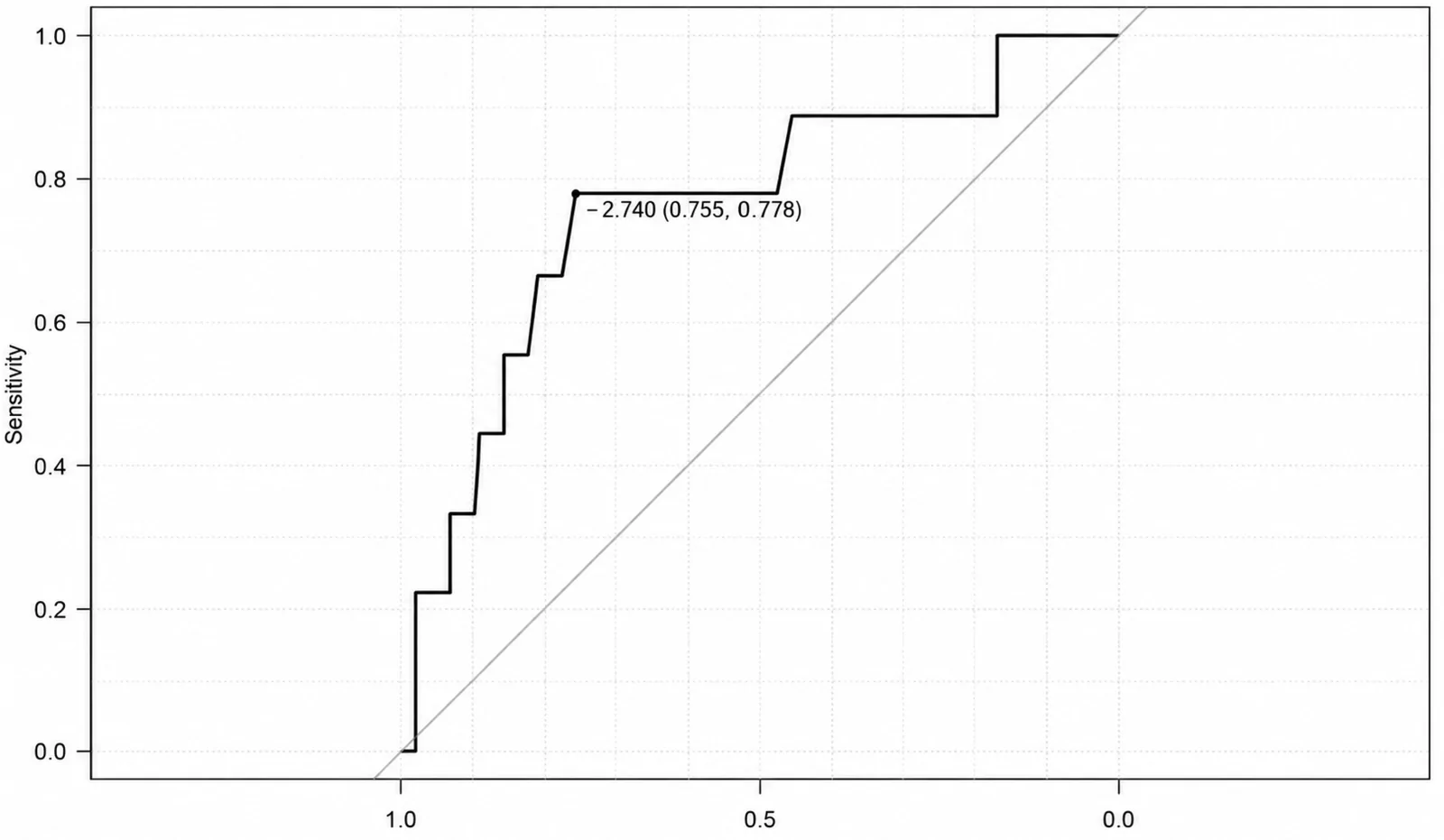
ROC curve analysis of preoperative ALBI score in predicting excessive postoperative bleeding (>1000 mL).

ROC curve analysis demonstrated discriminative performance for the preoperative ALBI score in predicting excessive postoperative bleeding exceeding 1000 mL, with an area under the curve (AUC) of 0.762 (95% CI: 0.581–0.944, *p* = 0.007) (Figure 3).

## 4. Discussion

This study evaluated the prognostic utility of the preoperative ALBI score in individuals undergoing isolated CABG. Our results demonstrated that a higher preoperative ALBI score was significantly associated with an increased postoperative drainage volume, an increased need for transfusions, and a prolonged mechanical ventilation duration. Furthermore, the ALBI score was a significant independent predictor of excessive postoperative bleeding exceeding 1000 mL.

The ALBI score was first introduced as a straightforward metric for assessing hepatic reserve in hepatocellular carcinoma cases using only two readily available laboratory parameters: serum albumin and total bilirubin [7]. Beyond its initial oncological application, accumulating evidence has highlighted its prognostic value across a range of cardiovascular conditions. Matsue et al. demonstrated that higher ALBI scores were independently associated with worse clinical outcomes in patients with acute heart failure [8]. Similarly, Duman et al. reported that the ALBI score can be used as a mortality predictor in valve surgery [9], while Yildirim et al. determined its predictive power for 30-day mortality after transcatheter aortic valve implantation [10]. To our knowledge, this study is the first to investigate the relationship between preoperative ALBI score and postoperative bleeding outcomes specifically in patients undergoing isolated CABG.

The underlying reason behind this relationship is the central role played by the liver in homeostasis [13]. The main contribution of this study is to demonstrate that even subclinical impairment in liver function can be associated with measurable differences in postoperative bleeding. Patients with clinically significant liver disease were excluded from our study and thus none of our patients had this condition. This suggests that the ALBI score detects a hemostatic vulnerability that conventional clinical assessment may fail to detect. Moreover, patients in the High ALBI group demonstrated significantly greater transfusion requirements and prolonged mechanical ventilation durations and required longer hospital stays. These findings are consistent with the known effects of excessive bleeding after surgery [14]. From a clinical perspective, the ALBI score offers several practical advantages as a preoperative risk stratification tool. It is calculated from two routinely available laboratory parameters, requires no additional testing, and can be easily integrated into standard preoperative workup without added cost. Identifying patients at high risk of bleeding before surgery allows surgeons to implement preventive strategies, for example, improving their nutritional status and carefully adjusting the timing of discontinuation of antiplatelet and anticoagulant medications [15]. It also enables the pre-determination of the hemostatic medication to be used in the postoperative period, helping to reduce the need for blood transfusions and related complications.

To date, several risk assessment models have been utilized to evaluate bleeding risk in cardiovascular cohorts [11,12]. Most of these existing tools primarily focus on renal function, baseline anemia, and antithrombotic regimens, often omitting subclinical hepatic impairment—a crucial factor in coagulation factor synthesis. The main contribution of our study is that it demonstrates that the ALBI score captures a distinct biological dimension of hemostatic vulnerability not fully addressed by traditional bleeding risk scores. Furthermore, as it relies on only two routinely available laboratory markers, it offers exceptional clinical practicality and cost-effectiveness.

From a pathophysiological perspective, it is highly likely that the preoperative ALBI score acts, at least in part, as an integrated indicator of subclinical organ dysfunction and underlying biological fragility. In advanced coronary artery disease, chronic systemic venous congestion and impaired cardiac output frequently lead to passive hepatic congestion and mild synthetic impairment. These changes manifest clinically as small decreases in serum albumin and transient increases in total bilirubin. While our multivariate analysis accounted for key procedural confounding factors such as cardiopulmonary bypass time, a high ALBI score likely reflects a range of subclinical hepatic fragility and latent coagulopathy that conventional risk classifiers, including EuroSCORE II, fail to capture. Furthermore, postoperative bleeding should be evaluated according to its timing. In the early postoperative period, surgical hemostasis and intraoperative anticoagulation, especially target and post-reversal ACT levels, are the main factors affecting blood loss [16]. As the effect of heparin decreases and coagulation returns to a stable state, the preoperative ALBI score may have a more lasting role in maintaining coagulation balance. However, our retrospective data did not include routine drainage measurements at 6 or 12 h, so we could not clearly compare early and late blood loss. We also recognize that the ALBI score is not a direct or primary cause of bleeding. Instead, it may serve as an additional marker of mild liver dysfunction that can affect bleeding risk together with surgical factors and anticoagulation management.

Another important finding was that patients in the High ALBI group had lower baseline hematocrit than those in the Low ALBI group. Baseline anemia is a known independent predictor of blood transfusion requirement and poor clinical outcomes after coronary artery bypass grafting [17]. Lower baseline hematocrit and higher ALBI scores may act synergistically, together reflecting a broader systemic state of biological fragility and reduced physiological reserve. While pre-existing anemia may contribute to the higher transfusion rates, subclinical liver dysfunction, as reflected by the ALBI score, may also help explain the increased postoperative drainage in these patients.

This study has several limitations. First, its retrospective and single-center design limits the generalizability of the findings. Second, the relatively small sample size and the low number of patients experiencing excessive bleeding exceeding 1000 mL (n = 9, 5.7%) represent a key limitation of our study. This small number of events resulted in wide confidence intervals for the calculated odds ratios in both univariate and multivariable logistic regression analyses. Third, dynamic changes in albumin and bilirubin levels during the perioperative period were not assessed. Furthermore, although patients with active severe infections were excluded and baseline inflammatory markers were comparable, the potential confounding effects of subclinical malnutrition or chronic catabolic states on serum albumin levels should be acknowledged as an unmeasured limitation. Fourth, patient classification was based on a sample-specific median cutoff score (−2.97) rather than an externally validated threshold. While using a median cutoff is a standard approach for exploratory continuous data analyses, this sample-derived threshold limits the direct generalizability of our findings to other patient populations. Fifth, secondary outcomes and subgroup analyses carry an inherent risk of false-positive findings due to multiple comparisons and should be interpreted as exploratory. Finally, while multivariable regression and ROC curve analysis (AUC = 0.762) demonstrated independent predictive value and good discrimination, we did not perform formal incremental reclassification or decision curve analyses against established clinical bleeding scores. Consequently, our findings demonstrate a strong statistical association rather than indicating the immediate clinical utility of this parameter. Future prospective studies with larger event counts are needed to formally assess whether adding the ALBI score to existing risk models yields a net improvement in reclassification in surgical decision-making.

## 5. Conclusions

In patients undergoing isolated coronary artery bypass grafting, a higher preoperative ALBI score was found to be independently associated with an increased risk of excessive postoperative bleeding. However, given the limited sample size, small absolute event count, and exploratory nature of the median cutoff, this finding represents an observational association rather than demonstrating clinical predictive utility. The preoperative ALBI score may serve as an additional biomarker to identify subclinical hepatic dysfunction and baseline risk, but prospective, large-scale multicenter studies are required to confirm these observations and validate its potential role in surgical decision-making.

## Figures and Tables

**Table 1:** Preoperative baseline characteristics according to ALBI group.

Variable	Low ALBI (n = 78)	High ALBI (n = 79)	*p*-Value
Age (years)	63.63 ± 10.95	61.10 ± 10.70	0.146
Female sex, n (%)	23 (29.5)	21 (26.6)	0.725
Body mass index (kg/m^2^)	27.67 ± 5.43	27.69 ± 4.80	0.981
Active smoking, n (%)	29 (37.2)	30 (38.0)	0.999
Diabetes mellitus, n (%)	40 (51.3)	36 (45.6)	0.524
Hypertension, n (%)	64 (82.1)	63 (79.7)	0.840
Hyperlipidemia, n (%)	77 (98.7)	74 (93.7)	0.210
Chronic obstructive pulmonary disease, n (%)	5 (6.4)	4 (5.1)	0.746
Chronic kidney disease, n (%)			0.280
Stage 1	41 (52.6%)	41 (51.9%)	
Stage 2	31 (39.7%)	26 (32.9%)	
Stage 3	5 (6.4%)	10 (12.7%)	
Stage 4	1 (1.3%)	0 (0.0%)	
Stage 5	0 (0.0%)	2 (2.5%)	
Peripheral arterial disease, n (%)	5 (6.4)	3 (3.8)	0.495
Carotid artery disease, n (%)	10 (12.8)	6 (7.6)	0.305
Left ventricular ejection fraction (%)	53.05 ± 10.14	52.46 ± 9.82	0.709
Previous coronary stenting, n (%)	15 (19.2)	14 (17.7)	0.840
Redo cardiac surgery, n (%) *	0 (0.0)	1 (1.3)	0.999
Urgent surgery, n (%)	3 (3.8)	7 (8.9)	0.328
EuroSCORE II	0.99 [0.71–1.47]	1.17 [0.79–2.27]	0.106
Low-molecular-weight heparin use, n (%)	11 (14.1)	11 (13.9)	0.999
ASA use, n (%)	61 (78.2)	63 (79.7)	0.847
P2Y12 inhibitor use, n (%)	28 (35.9)	35 (44.3)	0.330
Laboratory Parameters			
ALBI score	−3.29 ± 0.23	−2.61 ± 0.35	<0.001
Albumin (g/L)	44.92 ± 3.09	38.49 ± 4.02	<0.001
Total bilirubin (mg/dL)	0.48 [0.31–0.58]	0.62 [0.41–0.92]	<0.001
Hematocrit (%)	40.31 ± 4.88	38.22 ± 5.18	0.014
Creatinine (mg/dL)	0.90 [0.75–1.06]	0.93 [0.77–1.08]	0.744
Glomerular filtration rate	99.17 ± 39.86	92.00 ± 35.30	0.234
INR	1.03 (0.22)	1.16 (0.58)	0.061
ALT (U/L)	15.50 [12.00–22.00]	17.00 [12.00–24.50]	0.501
AST (U/L)	18.00 [15.00–25.00]	19.00 [14.00–22.00]	0.863
C-reactive protein (mg/L)	5.10 [2.02–12.20]	5.20 [2.20–13.70]	0.772
Calcium	9.31 ± 0.48	9.16 ± 0.44	0.042

ALBI, Albumin–Bilirubin; ASA, acetylsalicylic acid; ALT, alanine aminotransferase; AST, aspartate aminotransferase; INR, international normalized ratio. * The patient classified as undergoing redo cardiac surgery in the High ALBI group had previously undergone surgical repair of an atrial septal defect (ASD) but had no history of prior CABG.

**Table 2:** Postoperative outcomes and clinical characteristics according to ALBI group.

Variable	Low ALBI (n = 78)	High ALBI (n = 79)	*p*-Value
Acute kidney injury *dialysis* *non-dialysis*	1 (1.3) 20 (25.6)	1 (1.3) 17 (21.5)	0.785
Low cardiac output syndrome	14 (17.9)	15 (19.0)	0.999
ECMO support	0 (0.0)	2 (2.5)	0.497
Gastrointestinal complications	1 (1.3)	1 (1.3)	0.999
Late cardiac tamponade	1 (1.3)	1 (1.3)	1.000
IABP requirement	6 (7.7)	10 (12.7)	0.430
30-day mortality	2 (2.6)	7 (8.9)	0.167
Neurological events	1 (1.3)	3 (3.8)	0.620
Re-exploration for bleeding	2 (2.6)	3 (3.8)	0.999
Septic shock	0 (0.0)	1 (1.3)	0.999
Respiratory complications	10 (12.8)	12 (15.4)	0.819
Arrhythmia	24 (30.8)	19 (24.1)	0.371
Myocardial infarction	2 (2.6)	3 (3.8)	0.999
Mechanical ventilation time (hours)	7.00 [5.75–10.00]	10.00 [7.00–13.75]	<0.001
Total ERT (units)	1.00 [0.00–2.00]	2.00 [1.00–3.00]	0.001
Total FFP transfusion (units)	2.00 [1.00–2.00]	2.00 [1.00–4.00]	0.034
Total drainage volume (mL)	350.00 [300.00–487.50]	450.00 [350.00–700.00]	0.007
ICU length of stay (days)	2.00 [2.00–3.00]	2.00 [2.00–4.00]	0.159
Hospital length of stay (days)	7.00 [6.00–7.00]	7.00 [6.00–10.00]	0.003

AKI, acute kidney injury; ECMO, extracorporeal membrane oxygenation; IABP, intra-aortic balloon pump; ERT, erythrocyte transfusion; FFP, fresh frozen plasma; ICU, intensive care unit.

**Table 3:** Univariate and multivariate logistic regression analyses for predictors of postoperative bleeding >1000 mL.

	Univariate		Multivariate	
Variable	Odds Ratio (95% CI)	*p*-Value	Odds Ratio (95% CI)	*p*-Value
ALBI Score (per 1-unit increase)	5.93 (1.63–21.60)	0.0069	7.80 (1.87–32.50)	0.0048
ASA Use	1.07 (0.21–5.41)	0.936		
Gender (Male)	0.29 (0.07–1.13)	0.0746		
EuroSCORE II	1.04 (0.98–1.11)	0.213		
CPB Duration (per 1-min increase)	1.01 (1.00–1.03)	0.029	1.01 (0.999–1.03)	0.0633
Hypertension	2.22 (0.52–9.45)	0.28		

OR: odds ratio, CI: confidence interval, ALBI: Albumin–Bilirubin, CPB: cardiopulmonary bypass. Odds ratios for the ALBI score and CPB duration represent the risk increase per 1-unit and 1-min increase in the score, respectively.

## Data Availability

The data used and/or analyzed during the current study are not publicly available due to patient privacy and ethical restrictions but are available from the corresponding author on reasonable request.

## Abbreviations

The following abbreviations are used in this manuscript:

ACT	Activated clotting time
AKI	Acute kidney injury
ALBI	Albumin–Bilirubin
ALT	Alanine aminotransferase
ASA	Acetylsalicylic acid
ASD	Atrial septal defect
AST	Aspartate aminotransferase
AUC	Area under the curve
CABG	Coronary artery bypass grafting
CI	Confidence interval
COPD	Chronic obstructive pulmonary disease
CPB	Cardiopulmonary bypass
ECMO	Extracorporeal membrane oxygenation
ERT	Erythrocyte transfusion
FFP	Fresh frozen plasma
IABP	Intra-aortic balloon pump
ICU	Intensive care unit
INR	International normalized ratio
IQR	Interquartile range
LVEF	Left ventricular ejection fraction
OR	Odds ratio
ROC	Receiver operating characteristic
